# Atypical Charles Bonnet syndrome secondary to frontal meningioma: a case report

**DOI:** 10.1186/s12888-021-03360-6

**Published:** 2021-07-22

**Authors:** Lomelín-López Diana, Jaime Carmona-Huerta, J. Guillermo Patiño, Aldana-López Alejandro, Durand-Arias Sol

**Affiliations:** 1 Instituto Jalisciense de Salud Mental SALME, Zapopan, Jalisco Mexico; 2 Centro Universitario de Ciencias de la Salud, Universidad de Guadalajara, Guadalajara, Jalisco Mexico; 3 Instituto Nacional de Psiquiatría Ramón de la Fuente Muñiz, Ciudad de México, Mexico

**Keywords:** Charles-bonnet syndrome, Case report, Brain tumor, Meningioma, Visual hallucinations

## Abstract

**Background:**

Charles Bonnet Syndrome (CBS) is a rare clinical entity that is classically composed of visual hallucinations in the context of an altered optic pathway with preservation of reality judgment. This case aims to present the association of visual hallucinations with complex alterations of the nervous structures adjacent to the visual pathway and an atypical clinical presentation, thus explaining the possible mechanisms involved in the generation of these symptoms.

**Case presentation:**

A 43-year-old man presents seeking care due to visual hallucinations with partial preservation of reality judgment and symptoms compatible with a major depressive disorder, including irritability and diminished hygiene habits. He has a history of complete gradual loss of vision and hyposmia. Due to poor treatment response during hospitalization, an MRI was obtained, which showed a frontal tumor lesion with meningioma characteristics adjacent to the olfactory groove and compression of the optic chiasm. He underwent surgical resection of the lesion, which remitted the psychotic symptoms, but preserving the visual limitation and depressive symptoms.

**Conclusions:**

The presence of visual hallucinations, without other psychotic features as delusions, is a focus of attention for basic structural pathologies in the central nervous system. Affection at any level of the visual pathway can cause CBS. When finding atypical symptoms, a more in-depth evaluation should be made to allow optimization of the diagnosis and treatment.

## Background

Charles Bonnet Syndrome (CBS) is an infrequent entity that is often confused or misdiagnosed with other psychiatric pathologies and is classically observed in elderly patients with chronic visual deficits [1]. It was first described in 1760 by the Swiss scientist Charles Bonnet, who published an essay on the visual hallucinations his grandfather experienced after undergoing cataract surgery. Despite this, it was not until 1936 when the neurologist George de Morsier coined the term “Charles Bonnet Syndrome” [2].

There is no consensus for its definition, however, it is agreed that it presents with visual hallucinations in patients with previous alterations of the optic pathway, characteristically preserving the awareness of the reality, either partially or totally [3, 4]. By preserving the judgment of these visual abnormalities, it is said that one is facing the phenomenon called hallucinosis [3].

There are few publications in the scientific literature that focus on CBS. As of July 2020, 449 articles in Pubmed contained the words “Charles Bonnet” in the title or abstract, only 9 articles combined the words “brain tumor and hallucinations” and no article was found regarding brain tumor and CBS. Thus, the purpose of this article is to make a detailed description of an adult man with a brain tumor and CBS. This case report follows the CARE Guidelines [5].

## Case presentation

We present the case of a 43-year-old man who attended the outpatient service of a Psychiatric Hospital in Mexico, with a nine-month evolution of complex visual and auditory hallucinosis. He presents with bilateral amaurosis, which began insidiously 4 years prior, with no associated triggers and no previous studies to identify the cause. Psychiatric family history was denied.

He began his current condition with persistent visual and auditory hallucinosis, with a predominance of the former. He referred seeing his previous partner, from whom he had separated two and a half years prior and whom he had no contact, adding that she visited him at his house, would sit on his bed and talk to him. These episodes lasted several minutes and occurred continuously throughout the day, occurring more frequently in low-light environments. It is worth mentioning that he was fully aware that this phenomenon was not real and he referred to it as part of a disease, however, it caused him discomfort and sometimes agitation.

After the complete vision loss he initiated with depressive symptoms which consisted of sadness, anhedonia, fatigue, weight loss and low self-esteem, later adding recurrent thoughts of death. Family members mentioned that he began with personality changes such as constant irritability, impulsivity and behavioral changes regarding personal hygiene and dressing, lasting weeks without taking a bath, a situation that had never happened before. For this reason, he was taken to a psychiatric evaluation and was hospitalized for diagnosis and treatment, initially admitted with an unspecified non-organic psychotic disorder.

During the initial assessment, an ophthalmological and neurological alteration was found, being unable to perceive light in both eyes, with preserved eye movements, both slightly mydriatic pupils and bilateral hyposmia. The photomotor and the consensual light reflex were conserved, and the accommodation reflex was abolished. The rest of the physical exam was normal. He denied the presence of any specific visual complaint except for the vision loss. He also denied any physical symptoms like headaches, nausea or dizziness. Unfortunately, before his admission, he had not visited an ophthalmologist or neurologist for a check-up regarding the visual loss.

**Fig. 1:**
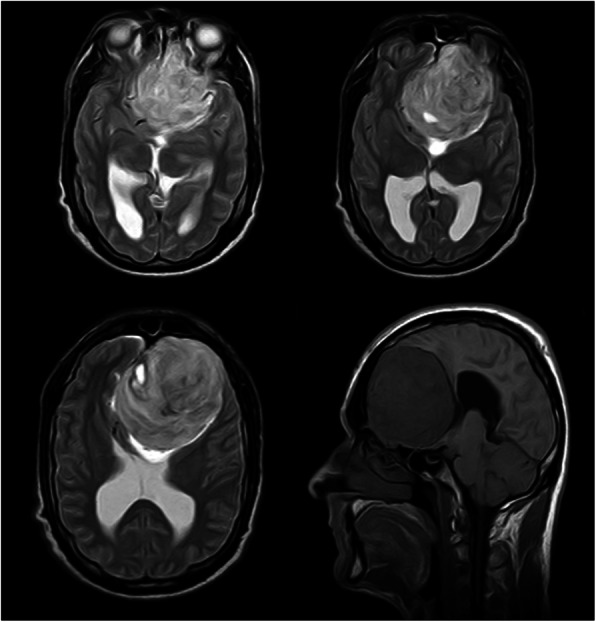
**a**, **b**, **c** Axial T2 brain MRI images showing a predominantly left frontal tumor lesion, with compression of the optic chiasma; **d**) Sagittal T1 brain MRI image showing frontal tumor lesion and compression of the anterior skull base structures

**Fig. 2:**
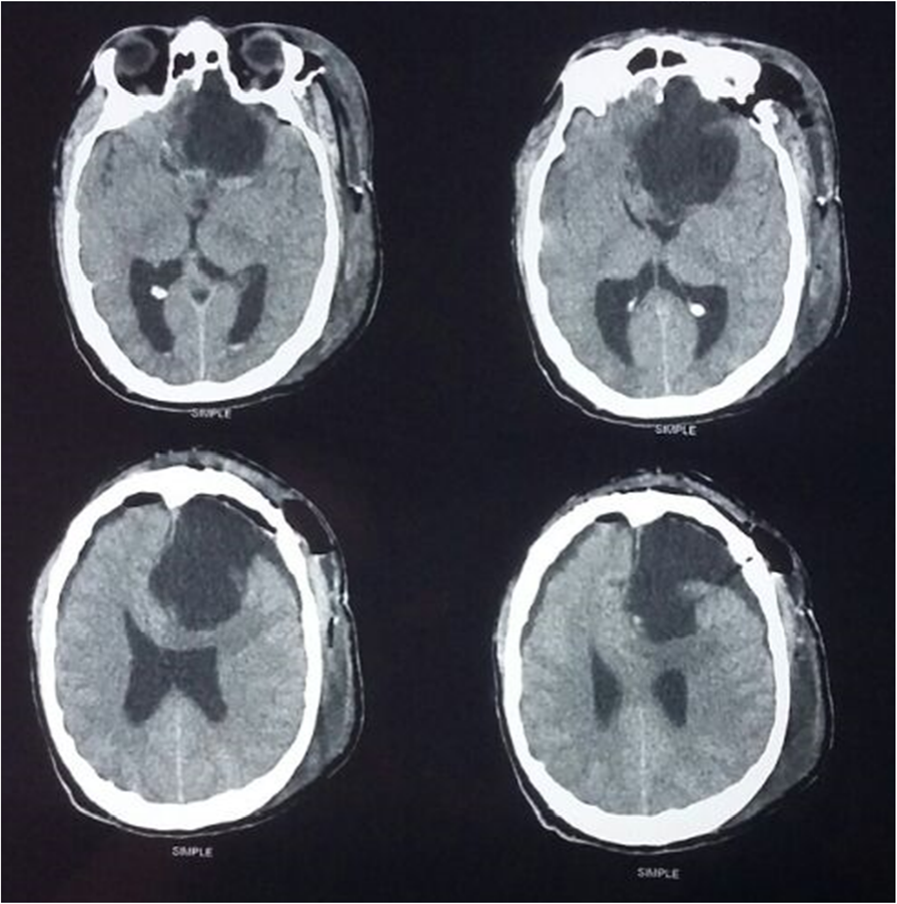
Postoperative non enhanced CT scan image showing axial cut after tumor resection with cavity occupied by cerebrospinal fluid (performed two months after the patient was discharged from the Psychiatric Hospital)

During his stay, the patient continued to present complex visual and auditory hallucinosis, referring that his ex-partner spoke to him and constantly warned him about his health, visiting him frequently in the hospitalization area: “Today I saw her. I was taking a bath and when I closed my eyes she told me to get ready, that something big was going to happen to me. I asked her what and she did not answer… I know that what I see is not real, it is a product of my mind, I know that I am not crazy … “ (sic patient). These symptoms did not ameliorate with the pharmacological treatment, which consisted of 3 mg of risperidone, 50 mg of sertraline and 0.25 mg of alprazolam every 24 h during 4 weeks. No neuropsychological tests were performed during his stay. Laboratory studies did not report abnormalities. The MRI revealed an occupational mass located at the anterior floor level of the skull adjacent to the olfactory groove in contact with the base of the sphenoid, with a mass effect on adjacent structures and significant compression of the optic chiasm, with characteristics suggestive of a benign meningioma (Fig. 1). He was referred to the neurosurgery department where a partial tumor resection was carried out by craniotomy with the confirmation of the pathology report of a benign meningioma (WHO Grade I) (Fig. 2). After the intervention, he achieved remission of the perception disturbances; however the visual impairment continued, which worsened the depressive symptoms. In addition to an exacerbation on impulsivity and low frustration tolerance, he carried out multiple suicide attempts, thus requiring six subsequent psychiatric hospitalizations. Information regarding his last follow- up reported him as stable, though apathetic and without senso-perceptive alterations. Neurologically he was reported with psychomotor retardation and frontal release signs. He remained unemployed and under his sister’s care.

## Discussion

Charles Bonnet Syndrome is characterized by visual hallucinations and is usually associated with visual pathway lesions. There is no known exact prevalence, this is mainly due to the multiple disagreements to define the criteria of the syndrome, however, prevalence’s that vary from 0.8% to 10 to 15% have been reported in patients with some alteration of the visual pathway. It is estimated that up to 0.6% of older adults present this condition [6, 7]. Hallucinations come on suddenly and can be simple or complex. The simple ones are usually composed of flashes of light, figures with simple contours and squared or branched patterns; while complex ones are vivid, elaborate and repetitive; the most frequent correspond to people, faces, animals, plants and objects [1, 3]. Although characteristically the alterations are usually visual, a variant is also known in which auditory alterations occur concomitantly [4, 8].

The most accepted theory regarding the hallucinatory phenomenon is known as the Sensory Deprivation Theory and explains how the lack of visual stimuli as a consequence of an injury or deficit causes an activation of the visual cortex by a change in its excitability, thus creating the characteristic hallucinations [1, 3, 6, 9, 10].

Charles Bonnet Syndrome can occur with any condition that causes affection at some point of the visual pathway, being intracranial tumors an important but scarcely reported neurological cause [11]. Because of the slow growth, brain tumors can remain neurologically silent and only present with psychiatric symptoms, with a prevalence ranging from 50 to 90% [12, 13]. They can cause a wide variety of neuropsychiatric symptoms, specifically when they develop in the frontal lobe [11]. It has been reported that 1/1000 of hospitalized psychiatric patients have brain tumors [14].

Within intracranial tumors, meningiomas have been frequently associated with psychiatric manifestations when they are located, in particular, on the right side of the frontal lobe, classically observing changes in personality, mood disorders and visual hallucinations, being the less common the auditory hallucinations [11]. It has been found that benign meningiomas exhibit psychiatric symptoms in approximately 21% of the patients, with no neurological or physical signs [14].

Meningiomas are the most common primary intracranial tumors, 80% of which are benign, with 1–5% localized in the medial anterior skull base. The predominant histology of those tumors is meningothelial and are linked to mutations in SMO gene, locus 7p32.1 [15]. Due to the location of the tumor, this type of benign meningioma is suspected, however, genetic tests were not performed.

Alterations at the frontal level causes dysfunction on reality judgment and an inadequate modulation of the fronto-limbic system that limits the adaptation to reality, metacognition processes and impulse control [16]. Once the etiology is known, it is necessary to assess whether the clinical features have at least one of the five characteristics that define atypical CBS: impaired introspection, various types of hallucinations, atypical psychological reaction to the situation, cognitive impairment and concomitant psychiatric disorder. In the case presented, the patient presented complex auditory and visual hallucinosis and a severe major depressive disorder, the most common comorbidity. Psychotic symptoms remitted after surgery, which rules out that there was a consequence of the depressive disorder, however, the latter condition persisted and subsequently worsened. The presence and localization of the meningioma explains the modification of the personality and behavioral changes.

## Conclusions

Charles Bonnet Syndrome should be suspected in the presence of visual perceptual alterations in patients with any previous condition that causes affection at some point of the visual pathway and who have an adequate awareness of the reality of the situation. In these cases, a joint initial psychiatric and neurological evaluation should be considered, because one of the causes, important and scarcely reported, are intracranial tumors. The atypical features are related with a worse prognosis due the difficulty of the diagnosis and the presence of poor insight and comorbidities. Close monitoring is important in similar cases since concomitant psychiatric disorders are frequent and there is a high risk of complications due to lack of timely and adequate diagnosis and treatment. Middle aged patients with new-onset psychiatric symptoms, sudden changes in personality, behavior or cognitive abilities need to be carefully studied; it is essential to search for focal or generalized neurologic symptoms, perform brain imaging and make a thorough neurological assessment [12–14].

## Data Availability

Data sharing is not applicable to this article as no datasets were generated or analyzed during the current study.

## Abbreviations

CBS: Charles Bonnet syndrome
mg: Milligrams
MRI: Magnetic resonance imagining
